# Telmisartan Attenuates Colon Inflammation, Oxidative Perturbations and Apoptosis in a Rat Model of Experimental Inflammatory Bowel Disease

**DOI:** 10.1371/journal.pone.0097193

**Published:** 2014-05-15

**Authors:** Hany H. Arab, Muhammad Y. Al-Shorbagy, Dalaal M. Abdallah, Noha N. Nassar

**Affiliations:** 1 Department of Biochemistry, Faculty of Pharmacy, Cairo University, Cairo, Egypt; 2 Department of Pharmacology and Toxicology, Faculty of Pharmacy, Cairo University, Cairo, Egypt; Vanderbilt University School of Medicine, United States of America

## Abstract

Accumulating evidence has indicated the implication of angiotensin II in the pathogenesis of inflammatory bowel diseases (IBD) via its proinflammatory features. Telmisartan (TLM) is an angiotensin II receptor antagonist with marked anti-inflammatory and antioxidant actions that mediated its cardio-, reno- and hepatoprotective actions. However, its impact on IBD has not been previously explored. Thus, we aimed to investigate the potential alleviating effects of TLM in tri-nitrobenezene sulphonic acid (TNBS)-induced colitis in rats. Pretreatment with TLM (10 mg/kg p.o.) attenuated the severity of colitis as evidenced by decrease of disease activity index (DAI), colon weight/length ratio, macroscopic damage, histopathological findings and leukocyte migration. TLM suppressed the inflammatory response via attenuation of tumor necrosis factor-α (TNF-α), prostaglandin E_2_ (PGE_2_) and myeloperoxidase (MPO) activity as a marker of neutrophil infiltration besides restoration of interleukin-10 (IL-10). TLM also suppressed mRNA and protein expression of nuclear factor kappa B (NF-κB) p65 and mRNA of cyclo-oxygenase-2 (COX-2) and inducible nitric oxide synthase (iNOS) proinflammatory genes with concomitant upregulation of PPAR-γ. The alleviation of TLM to colon injury was also associated with inhibition of oxidative stress as evidenced by suppression of lipid peroxides and nitric oxide (NO) besides boosting glutathione (GSH), total anti-oxidant capacity (TAC) and the activities of superoxide dismutase (SOD) and glutathione peroxidase (GPx). With respect to apoptosis, TLM downregulated the increased mRNA, protein expression and activity of caspase-3. It also suppressed the elevation of cytochrome c and Bax mRNA besides the upregulation of Bcl-2. Together, these findings highlight evidences for the beneficial effects of TLM in IBD which are mediated through modulation of colonic inflammation, oxidative stress and apoptosis.

## Introduction

Inflammatory bowel diseases (IBD), including ulcerative colitis (UC) and Crohn’s disease (CD), are chronic, relapsing, immunologically mediated inflammatory disorders of the gastrointestinal tract that jeopardize the quality of life of patients suffering from these disorders [1]. During the progression of IBD, disruption of intestinal epithelial barrier is regarded as the central event in IBD pathogenesis which is followed by robust immune responses towards intestinal flora in a context of genetic predisposition [2]. Activation of intestinal immune system is associated with excessive generation of inflammatory cytokines such as tumor necrosis factor-α (TNF-α) which amplifies the inflammatory cascade by triggering the generation of other proinflammatory cytokines and enhancing the recruitment of macrophages and neutrophils [1], [2]. The infiltration of neutrophils generates excessive amounts of reactive oxygen species (ROS), nitric oxide (NO) and prostaglandin E_2_ (PGE_2_) which ultimately provoke mucosal disruption [1]. Excessive generation of ROS and cytokines has been reported to activate several transcription factors that upregulate the inflammatory response. Among them, the nuclear factor kappa B (NF-κB) induces transcription of proinflammatory genes including cyclo-oxygenase-2 (COX-2) and inducible nitric oxide synthase (iNOS) [3]. Increased levels of interleukin-10 (IL-10) have been reported in IBD patients [4] and experimental animals [5], [6] where they attenuate the exaggerated inflammatory response [2]. The pathogenesis of IBD also involves increased frequency of apoptosis with consequent loss of intestinal epithelial cells [7].

Angiotensin II (Ang II), the main effector peptide of the rennin-angiotensin system (RAS), has potent proinflammatory features linked with the pathogenesis of several chronic inflammatory disorders including IBD [8]. Via its actions on angiotensin II type 1 (AT1) receptors, angiotensin II promotes tissue inflammation through upregulation of adhesion molecules, increasing vascular permeability, and thus, enhancing neutrophil infiltration, which contributes to gut ulceration [9]. It also increases the release of proinflammatory cytokines such as TNF-α, probably, through activation of NF-κB. Additionally, Ang II triggers oxidative stress via activation of NADH/NADPH oxidase with consequent generation of superoxide anions [8].

Accumulating evidence has indicated the efficacy of members of Ang II receptor blockers (ARBs) such as valsartan and olmesartan in the attenuation of colon injury in experimental colitis [10], [11]. Among several candidates of ARBs, telmisartan (TLM) has unique anti-inflammatory and antioxidant features owing to the blockade of Ang II AT1 receptors besides its partial agonist actions on peroxisome proliferator activated receptor-gamma (PPAR-γ) [12]. Previously, PPAR-γ agonists such as rosiglitazone have displayed marked protective effects in experimental colitis [13]. Interestingly, TLM has exerted versatile beneficial effects against atherosclerosis and myocardial infarction [14], [15]. TLM also exhibits favorable actions in vascular dysfunction [16], cardiac remodeling [17], renal injury [18], hepatic fibrosis [19], stroke [20] and testicular injury [21]. Additional advantages of TLM include excellent toxicity profile, the longest half-life among all ARBs, and its cost-effective price [12]. Together, these findings encouraged us to investigate the potential alleviating effects of TLM and the underlying mechanisms in tri-nitrobenezene sulphonic acid (TNBS)-induced colitis, an experimental model of human IBD.

In the current study, colon inflammation was assessed by disease activity index (DAI), colon weight/length ratio, macroscopic damage, histopathological assessment and leukocyte invasion as indicated by myeloperoxidase (MPO) activity. To delineate the underlying mechanisms of TLM, we investigated its effects on the inflammatory status by assessing the mRNA/protein expression of NF-κB together with the mRNA expression of COX-2, iNOS and PPAR-γ. Besides, the colonic levels of TNF-α, IL-10 and PGE_2_ were investigated. The redox status was monitored by assessing the levels of lipid peroxides, NO, reduced glutathione (GSH) and total antioxidant capacity (TAC) along with superoxide dismutase (SOD) and glutathione peroxidase (GPx) antioxidant enzymes. Additionally, we investigated the colonic apoptosis via estimating the mRNA expression of cytochrome c, Bcl-2 associated x protein (Bax) and B cell lymphoma-2 (Bcl-2) in addition to the mRNA, protein expression and the activity of caspase-3. To the best of our knowledge, this is the first report that describes the ameliorative effects of TLM in TNBS-induced colitis via its anti-inflammatory, anti-oxidant and anti-apoptotic actions.

## Materials and Methods

### Ethics Statement

This study was carried out in strict accordance with the recommendations in the Guide for the Care and Use of Laboratory Animals published by the US National Institute of Health (NIH publication No. 85-23, revised 1996). The protocol was approved by the Committee of Animal Care and Use of Faculty of Pharmacy, Cairo University. All efforts were made to minimize animal suffering.

### Animals

Adult male Wistar rats weighing 200±20 g were purchased from the National Institute for Research, Cairo, Egypt. The animals were kept at controlled environmental conditions in terms of constant temperature (23±1 °C), humidity (60±10%), and a 12/12 h light/dark cycle. They were acclimatized for one week before any experimental procedures and were allowed standard rat chow and water *ad libitum*.

### Chemicals

Telmisartan (Micardis) was obtained from Boehringer Ingelheim, Germany. TNBS was purchased from Sigma-Aldrich (St. Louis, MD, USA). All Other chemicals were of highest purity and analytical grade. ELISA kits for determination of TNF-α, IL-10 and PGE_2_ along with caspase-3 colorimetric kit were purchased from R &D systems, MN, USA while the TAC kit was provided by Cayman Chemical Company, Ann arbor, MI, USA.

### Experimental design and treatment protocol

In the current study, animals were randomly divided into four groups (8 rats per group). Group I (Control gp): received physiological saline rectally + oral vehicle. Group II (Control + TLM gp): received saline rectally + oral TLM. Group III (TNBS gp): received rectal TNBS instillation (50 mg/kg) + oral vehicle. Group IV (TNBS + TLM): received TNBS rectally + oral TLM. TLM was suspended in 0.5% carboxymethyl cellulose vehicle and was administered (10 mg/kg/day) by oral gavage starting 1 week before the induction of TNBS colitis and was continued till the 4^th^ day post TNBS instillation. The animals were euthanized using an overdose of anesthesia on the 5^th^ day of TNBS induction. The selected dose of TLM was based on its previously displayed anti-inflammatory actions in animal models of autoimmune myocarditis [22], cardiac ischemic reperfusion injury [23], myocardial infarction [15] and hepatic fibrosis [19]. The chosen regimen is consistent with previous reports investigating the effects of olmesartan and valsartan in experimental colitis [10], [11] and the PPAR-γ agonist rosiglitazone in TNBS colitis [13].

### Induction of colitis

TNBS colitis was induced according to the procedures described by Morris et al. [24] with modifications [25]. Briefly, animals were fasted for 24 hours with free access to water. Animals were anaesthetized with chloral hydrate (300 mg/kg i.p.) and a medial grade polyurethane catheter with 2 mm external diameter was inserted into the anus and its tip was advanced in the descending colon to 8 cm from the anus verge. Rats were kept in a vertical head-down position and TNBS (50 mg/kg) in 50% ethanol was rectally instilled slowly within 1 min and the catheter was kept in place for another min, and gently removed. Then, TNBS-treated rats were left in the head-down position for 1 min to avoid leakage of the intracolonic instillate and then kept on warm bedding till regain of consciousness. The control group received physiological saline rectally instead of TNBS solution.

### Tissue collection and preparation

On the 5^th^ day post TNBS-instillation, rats were euthanized under deep ether anesthesia and laparatomy was immediately performed. The distal 8 cm portion of the colon was excised, freed of adherent adipose tissue, longitudinally split and washed with ice-cold saline to remove fecal residues. Then it was blotted dry, weighed and macroscopic assessment of colitis was performed. Sections of the distal colon were utilized for histopathological, immunohistochemical and biochemical investigations.

### Measured parameters

#### 1. Disease activity index (DAI)

The scores of DAI ranging from 0 (healthy) to 12 (severe colitis) were calculated as previously described [26]. The sum of scores for the % loss of body weight (score 0-4), stool consistency (score 0-4) and rectal bleeding (scores 0-4) were calculated (Table 1). Diarrhea was manifested by presence of mucus on animal feces sticking to fur while rectal bleeding ranged from occult blood to gross bleeding on the fecal matter.

**Table 1:** Scoring of disease activity index (DAI).

Weight loss	Stool consistency	Rectal bleeding
0 = < 1%	0 = normal	0 = negative
1 = 1-5%	2 = loose stool	2 = positive
2 = 5-10%	4 = diarrhea	4 = gross bleeding
3 = 10-15%		
4 = >15%		

#### 2. Assessment of colon damage by macroscopic scoring

The severity of colitis was evaluated by an independent observer blinded to the identity of treatments. The colon damage was scored on a 0–10 scale according to the criteria described by Tsune et al. [27]: 0  =  no macroscopic changes; 1 =  focal hyperemia, no ulcers; 2  =  ulcer without significant inflammation (hyperemia and bowel wall thickening); 3  =  ulceration with inflammation at one site; 4  =  two or more sites of ulceration/inflammation; 5  =  major sites of damage extending > 1cm along colon length; 6 – 8  =  when the area of damage exceeds 2 cm along the colon, the score was increased by one for each additional 1 cm. Besides, the adhesion scores were added according to the criteria of Bobin-Dubigeon et al. [28]: 0  =  no adhesion; 1  =  minor adhesion; 2  =  major adhesion.

#### 3. Histopathological examination and microscopic scoring

Full thickness colon biopsy specimens were fixed in 10% buffered formol saline for 24 h. The specimens were washed, dehydrated by alcohol, cleared in xylene and embedded in paraffin at 56°C in hot air oven for another 24 h. Sections of 3 µm thickness were stained with hematoxylin and eosin (H&E) and examined under the light microscope (Leica Microsystems, Germany). All histopathologic processing and assessment of specimens were performed by an experienced observer blinded to the identity of the sample being examined to avoid any bias.

The colon microscopic damage was scored on a 0–5 scale as described by Galvez et al. [29] as follows: 0  =  normal colonic tissue; 1  =  inflammation or focal ulceration limited to the mucosa; 2  =  focal or extensive ulceration and inflammation limited to the mucosa and the submucosa; 3, focal or extensive ulceration and inflammation with involvement of muscularis ; 4  =  focal or extensive ulceration and inflammation with involvement of the serosa; and 5, extensive ulceration and transmural inflammation with involvement of the serosa.

#### 4. Immunohistochemical detection of NF-κB p65 and caspase-3

Paraffin embedded tissue sections of 3 µm thickness were rehydrated in xylene and then in graded ethanol solutions and heated in citrate buffer (pH 6) for 5 min. The slides were blocked with 5% bovine serum albumin (BSA) in Tris buffered saline (TBS) for 2 h. The sections were then immunostained with primary polyclonal rabbit anti-NF-κBp65 (Santa Cruz Biotechnology Inc, CA, USA) or caspase-3 (Thermo Scientific, IL, USA) at a concentration of 1 µg/ml in 5% BSA in TBS and were incubated overnight at 4 °C. Following primary antibody incubation step, the slides were washed with TBS and were then incubated with goat anti-rabbit secondary antibody. Finally, the sections were washed with TBS and incubated for 5–10 min in a solution of 0.02% diaminobenzidine (DAB) containing 0.01% H_2_O_2_. Counter staining was performed using hematoxylin and the slides were visualized under light microscope (Leica Microsystems, Germany).

#### 5. Colon MPO activity

Activity of MPO, a marker for neutrophil infiltration, was estimated according to the method of Krawisz et al. [30] with slight modifications. One unit of MPO activity is defined as the amount of enzyme converting 1 µmol of H_2_O_2_ to water in 1 min at 25°C. The colon homogenates were subjected to 3 cycles of freezing/thawing, 30 sec of sonication and centrifuged at 20,000×g for 20 min at 4°C. O-dianisidine hydrochloride (0.167%) and H_2_O_2_ (0.0005%) in potassium phosphate buffer (50 mmol/L, pH 6) were added to the supernatant and the absorbance rate was monitored at 460 nm for 4 min.

#### 6. Inflammatory cytokines (TNF-α and IL-10)

The levels of TNF-α and IL-10 in colon homogenate supernatants were measured using ELISA kits (R &D systems incorporation, USA). All the procedures were performed according to the manufacturer's instructions. The assays of these cytokines employ the quantitative sandwich enzyme immunoassay technique and the optical densities were measured at 450 nm using microplate reader (Biochrom Asys, UK). The intensity of the color was proportional to the amount of the corresponding cytokine bound in the initial step. The corresponding levels were expressed as pg/g tissue.

#### 7. PGE_2_ concentration

The levels of colon PGE_2_ were determined using an ELISA kit (R &D systems incorporation, USA), according to the manufacturer's instructions and the colonic levels were presented as pg/ mg tissue.

#### 8. Lipid peroxides concentration

Determination of lipid peroxide levels, expressed as malondialdehyde (MDA), was carried out according to the thiobarbituric acid assay of Buege and Aust [31]. The absorbance was recorded at 535 nm and the results were expressed as nmol/g tissue.

#### 9. Nitric oxide concentration

Total NO was determined by measuring its stable metabolites, particularly, nitrite (NO_2_^−^) and nitrate (NO_3_^−^) based on the method of Miranda et al. [32] with the modification of replacing zinc sulfate instead of ethanol for the precipitation of proteins in the supernatant of colon homogenates. Absorbance was measured at 540 nm and the results were expressed as nmol/g tissue.

#### 10. Reduced glutathione

Colon GSH levels were determined as previously described by Beutler et al. [33], using 5,5’- dithiobis 2-nitrobenzoic acid (DTNB) reagent. The optical density for the colored product was read at 412 nm and results were expressed as nmol/g tissue.

#### 11. Determination of TAC

TAC was determined using Cayman total antioxidant assay kit according to the manufacturer’s instructions. The assay relies on the ability of antioxidants in the supernatants of colon homogenates to inhibit the oxidation of ABTS (2,2-azino-di-[3-ethylbenzthiazoline sulphonate]) by metmyoglobin. The amount of the oxidized product was estimated by reading absorbance at 405 nm. The capacity of the antioxidants in the sample to prevent ABTS oxidation was compared to that of Trolox, a water-soluble tocopherol analogue and the results were quantified as µmol of Trolox equivalent/g tissue.

#### 12. Superoxide dismutase activity

SOD activity was assayed according to the method of Marklund and Marklund [34] which assesses the ability of colonic SOD to prevent auto-oxidation of pyrogallol. The change in absorbance at 420 nm was obtained at 1 min interval for 3 min. One unit of SOD is defined as the amount of enzyme that affords 50% inhibition of pyrogallol auto-oxidation in 1 min. Results were expressed as U/mg protein. The protein content of colonic homogenate was determined using the method of Lowry et al. [35].

#### 13. Glutathione peroxidase activity

GPx activity was determined according to the method of Paglia and Valentine [36], that resides on the ability of the enzyme to oxidize GSH which was monitored via recording the decrease in absorbance of NADPH at 340 nm. One unit of enzyme is defined as the amount of enzyme that oxidizes 1 µmol NADPH /min at 25°C.

#### 14. Quantitative real-time RT-PCR

Total RNA was extracted from colon tissues using RNeasy Mini kit (Qiagen, CA, USA) and the purity of obtained RNA was verified spectrophotometrically at 260/280 nm. Equal amounts of RNA (2 µg) were reverse transcribed into cDNA using Superscript Choice systems (Life Technolgies, USA) according to the manufacturer’s instructions. To assess the expression of inflammation and apoptosis-associated target genes, quantitative real-time PCR was performed using SYBR green PCR Master mix (Applied Biosystems, CA, USA) as described by the manufacturer. Briefly, in a 25 µl reaction volume, 5 µl of cDNA was added to 12.5 µl of 2× SYBR green Master mix and 200 ng of each primer. The sequences of primers are described in Table 2. PCR reactions included 10 min at 95°C for activation of AmpliTaq Gold DNA polymerase, followed by 40 cycles at 95°C for 15 sec (denaturing) and 60°C for 1 min (annealing/extension). The expression level was calculated from the PCR cycle number (C_T_) where the increased fluorescence curve passes across a threshold value. The relative expression of target genes was obtained using comparative C_T_ (ΔΔC_T_) method. The ΔC_T_ was calculated by subtracting GAPDH C_T_ from that of target gene whereas ΔΔC_T_ was obtained by subtracting the ΔC_T_ of calibrator sample (control gp) from that of test sample (control+ TLM, TNBS or TNBS+ TLM gp). The relative expression was calculated from the 2^-ΔΔCT^ formula [37].

**Table 2:** Primer sequences used for real-time PCR.

mRNA species	Accession no.	Primer sequence
PPAR-γ	NM_013124	Forward 5’- AGACCACTCCCACTCCTTTG -3’
		Reverse 5’-AGGTCATACTTG TAATCTGC-3’
NF-κB p65	NM_199267	Forward 5’-GTCATCAGGAAGAGGTTTGGCT-3’
		Reverse 5’-TGATAAGCTTAGCCCTTGCAGC-3’
COX-2	NM_017232	Forward 5’-GCTCAGCC ATACAGCAAATCC-3’
		Reverse 5’-GGGAGTCGGGCAAT CATCAG-3’
iNOS	NM_ 012611	Forward 5’-ACCTTCCGGGCAGCCTGTGA-3’
		Reverse 5’-CAAGGAGGGTGGTGCGGCTG-3’
Cytochrome C	NM_012839	Forward 5’-TTTGAATTCCTCATTAGTAGCTTTTTTGG-3’
		Reverse 5’-CCATCCCTACGCATCCTTTAC-3’
Bax	NM_017059	Forward 5’-CAAGAAGCTGAGCGAGTGTCT-3’
		Reverse 5’-CAATCATCCTCTGCAGCTCCATATT-3’
Bcl-2	NM_016993	Forward 5’-TGCGCTCAGCCCTGTG-3’
		Reverse 5’-GGTAGCGACGAGAGAAGTCATC-3’
Caspase-3	NM_012922	Forward 5’-GCAGCTAACCTCAGAGAGACATTC-3’
		Reverse 5’-ACGAGTAAGGTCATTTTTATTCCTGACTT-3’
GAPDH	NM_017008	Forward 5’-TGCTGGTGCTGAGTATGTCG-3’
		Reverse 5’-TTGAGAGCAATGCCAGCC-3’

#### 15. Caspase-3 activity

Caspase-3 activity was colorimetrically assayed using R &D systems kit as described by the manufacturer. Briefly, an aliquot of the homogenate supernatant was incubated with the labeled substrate DEVD-pNA (acetyl-Asp-Glu-Val-Asp p-nitroanilide). The cleavage of the peptide by the caspase releases the chromophore pNA, which was read at 405 nm using Biochrom Asys microplate reader, UK. According to the manufacturer’s instructions, the results were expressed as fold change of caspase-3 activity.

### Statistical analysis

Parametric data were expressed as mean ± SEM, and statistical comparisons were carried out using one-way analysis of variance (ANOVA), followed by Tukey-Kramer post hoc test which was used for multiple comparisons between groups. Non-parametric values were expressed as median and the statistical differences among groups were identified using Kruskal-Wallis analysis of variance followed by the rank-based Mann–Whitney U-test for group comparisons. Statistical analysis was performed using SPSS program, version 17. The minimal level of significance was identified at p<0.05.

## Results

### Telmisartan ameliorates the severity of TNBS-induced injury in rats

**Figure 1:**
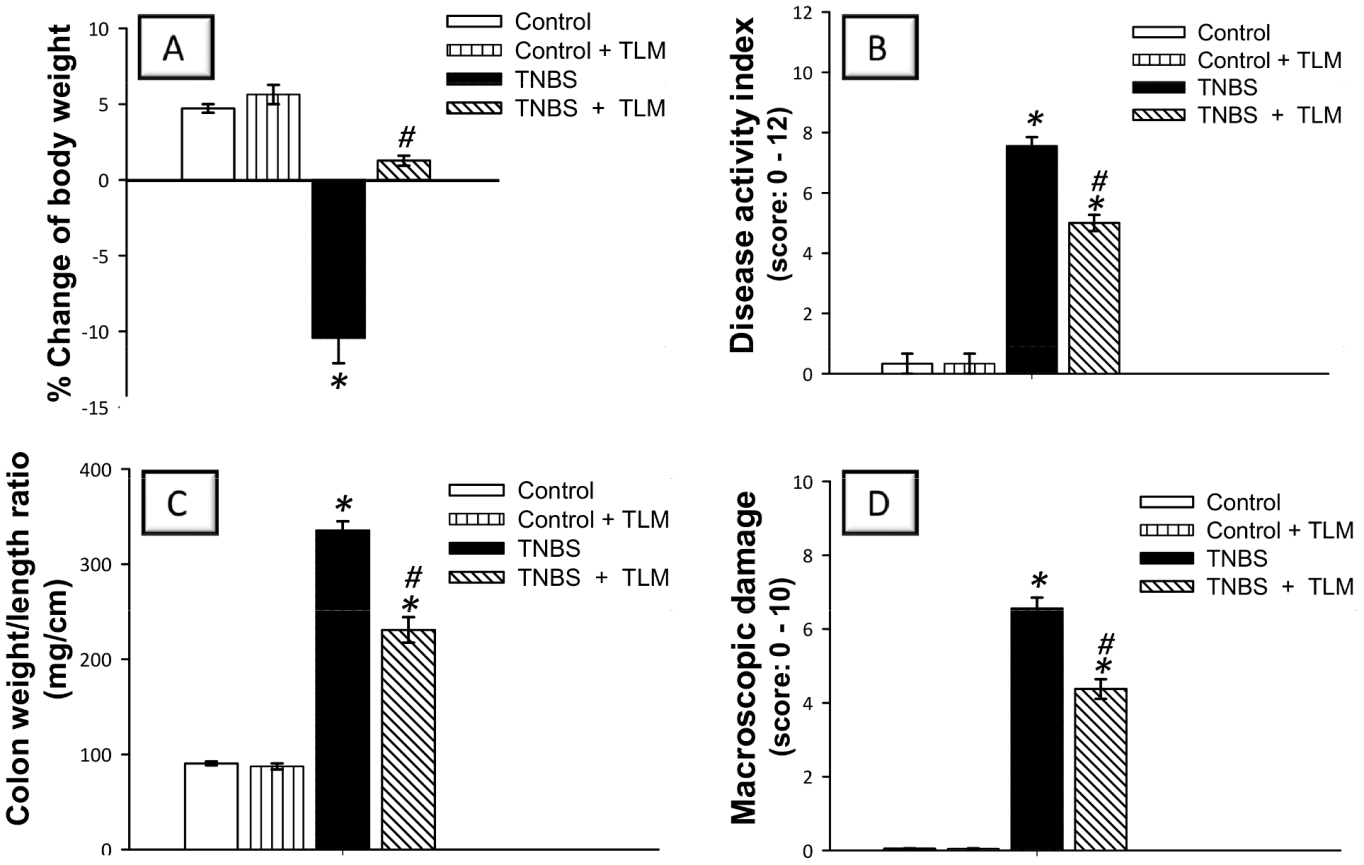
Effect of telmisartan on the severity of TNBS-induced colitis in rats. (A) Change of Body weight (%) (B) Disease activity index. (C) Colon weight/length ratio. (D) Colon macroscopic damage. Colon injury was induced by a single intrarectal instillation of TNBS (50 mg/kg) in 50% ethanol solution whereas the control group received the same volume of physiological saline solution rectally. Telmisartan was orally administered (10 mg/kg/day), starting 1 week before TNBS instillation and was continued till the 4^th^ day post TNBS insult. On the 5^th^ day, rats were euthanized and the colons were immediately excised. Values of body weight changes and colon weight/length ratio (parametric data) are expressed as mean ± SEM (n =  8) while the scores of disease activity index and macroscopic damage (non-parametric) are expressed as median; n = 8. *Significant difference from control gp at *p*<*0.05, #* Significant difference from TNBS colitis gp at *p*<*0.05.* TLM; telmisartan, TNBS; tri-nitrobenzene sulfonic acid.

We assessed the efficacy of TLM in alleviating colon injury using TNBS-induced colitis, an experimental model of human IBD [38]. To investigate the severity of colitis, its clinical signs including body weight loss, diarrhea and rectal bleeding were explored. Rats challenged with TNBS suffered marked weight loss (>10%) as a result of colonic inflammation compared with vehicle-treated control group (Figure 1A). The animals also displayed high DAI scores associated with incidence of diarrhea and rectal bleeding in addition to increased colon weight/length ratio, a reliable marker of colon inflammation [10], (Figure 1B, C). These data were confirmed by the macroscopic examination of colon that revealed severe colonic injury characterized by mucosal damage, thickening of bowel wall, hyperemia, edema and ulcerations (Figure 1D). Interestingly, TLM mitigated these changes and diminished the severity of colonic injury as compared to TNBS colitis group. Thus, these data suggest that TLM attenuated the development of TNBS colitis.

### Telmisartan mitigates colonic histopathological changes and recruitment of immuno-inflammatory cells

**Figure 2:**
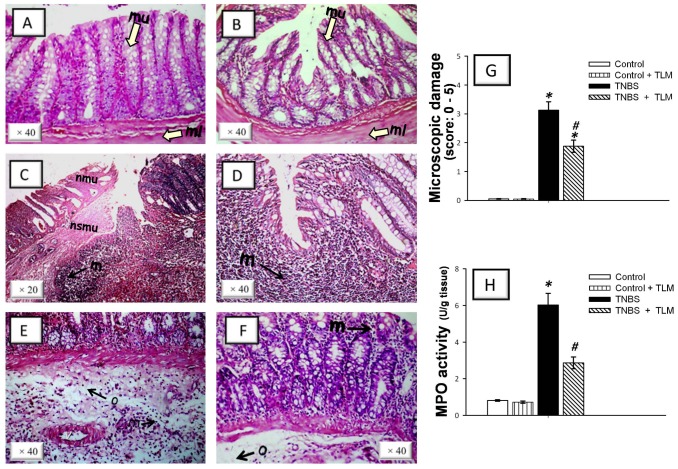
Telmisartan ameliorates histopathological damage and myeloperoxidase activity in colonic tissues of rats with TNBS colitis. Representative photomicrographs of sections from colonic samples taken on the 5^th^ day post TNBS-rectal instillation. (A) Control rats receiving saline rectally showed normal architecture of mucosa (mu) with intact epithelial surface, submucosa and muscularis (ml) layer (open arrows). (B) Control rats receiving saline rectally + TLM (10 mg/kg/day p.o.) displayed no histological modifications. (C-E) TNBS-treated group (50 mg/kg) was characterized by focal necrosis of mucosa (nmu) and submucosa (nsmu) and diffuse infiltration of leukocytes (m) and edema (o) in the submucosal layer. (F) TNBS + TLM administration (10 mg/kg/day p.o.) revealed mucosal preservation, diminished inflammatory cell invasion and edema. Hematoxylin and eosin staining. (G) Microscopic damage scores (expressed as median; n = 8). (H) TLM inhibits colon myeloperoxidase (MPO) activity (mean ± SEM; n = 8). Histological analysis was performed 5 days post TNBS instillation and TLM was administered for 12 days starting 1 week before colitis induction. *Significant difference from control gp at *p*<*0.05, #* Significant difference from TNBS colitis gp at *p*<*0.05.* TLM; telmisartan, TNBS; tri-nitrobenzene sulfonic acid.

We next assessed whether TLM has protective effects against the histopathological damage in colons of rats with TNBS colitis. Colon sections from control and control+TLM groups revealed an intact architecture of colon tissues (Figure 2A, B). On the other hand, colons of TNBS group revealed significant tissue injury with high scores of microscopic damage indicating focal necrosis of mucosa and submucosal and ulceration of the colonic mucosa with loss of lining epithelium. Diffuse leukocyte infiltration, mainly as neutrophils, was detected in the mucosa including the lamina propria, in addition to submucosa, muscularis. In addition, diffuse edema was observed in the submucosal layer (Figure 2C–E). TLM protected against these alterations and reduced the histopathological scores revealing attenuated inflammatory cell infiltration and preservation of colon cytoarchitecture while edema was still detected (Figure 2F, G).

Leukocyte invasion to colonic tissues was confirmed by a 7.4-fold increase of MPO activity, a biochemical index for neutrophil influx [39], as compared to the control group (Figure 2H). TLM administration afforded a 53% reduction of MPO activity as compared to TNBS group. Together, these data indicate that TLM attenuated mucosal damage and leukocyte invasion in TNBS-induced colitis.

### Telmisartan modulates colon inflammatory cytokines and PGE_2_

**Figure 3:**
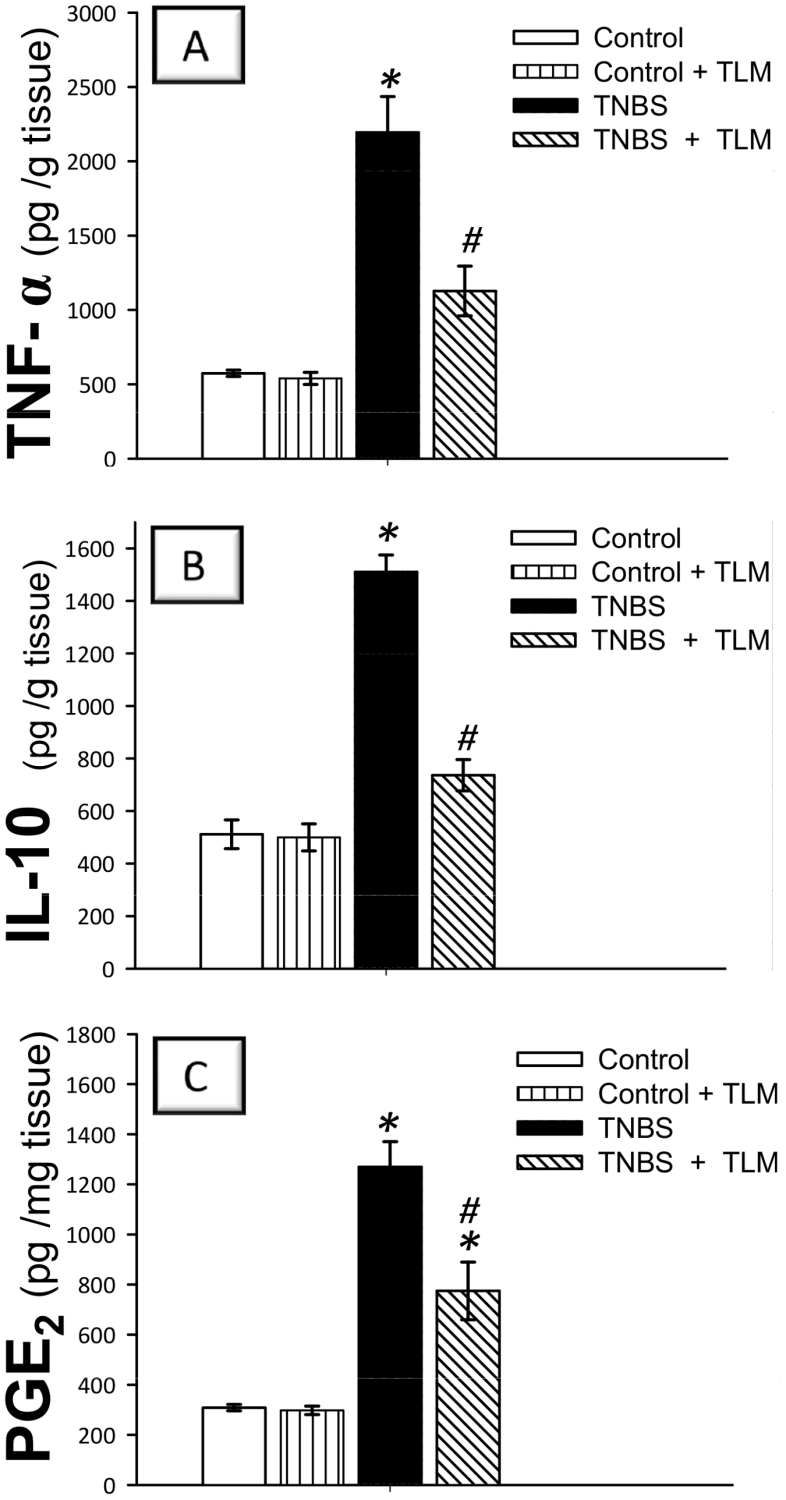
Telmisartan modulates inflammatory cytokines and PGE_2_.in colon of rats with TNBS colitis. Levels of tumor necrosis factor-α; TNF-α (A), interleukin-10; IL-10 (B) and prostaglandin E_2_; PGE_2_ (C) were determined by ELISA. Measurements were performed 5 days post TNBS instillation and TLM was administered for 12 days starting 1 week before colitis induction. Data are expressed as mean ± SEM (n =  8) *Significant difference from control gp at *p*<*0.05, #* Significant difference from TNBS colitis gp at *p*<*0.05.* TLM; telmisartan, TNBS; tri-nitrobenzene sulfonic acid.

To gain an insight into the inflammatory milieu of colons, we investigated the levels of TNF-α & IL-10 cytokines along with PGE_2_. Instillation of TNBS resulted in severe inflammatory response as indicated by remarkable increases in colonic levels of the proinflammatory TNF-α (380%) and PGE_2_ (412%) as compared to the control group (Figure 3A, C). Meanwhile, the anti-inflammatory IL-10 levels were also elevated (295% of control group). Administration of TLM lowered TNF-α, PGE_2_ and IL-10 by 49%, 39% and 51% respectively, as compared to TNBS colitis group. These observations indicate that TLM can modulate the inflammatory cytokines and PGE_2_ to mitigate TNBS colitis.

### Telmisartan abrogates the mRNA expression of NF-κB, COX-2 and iNOS genes

**Figure 4:**
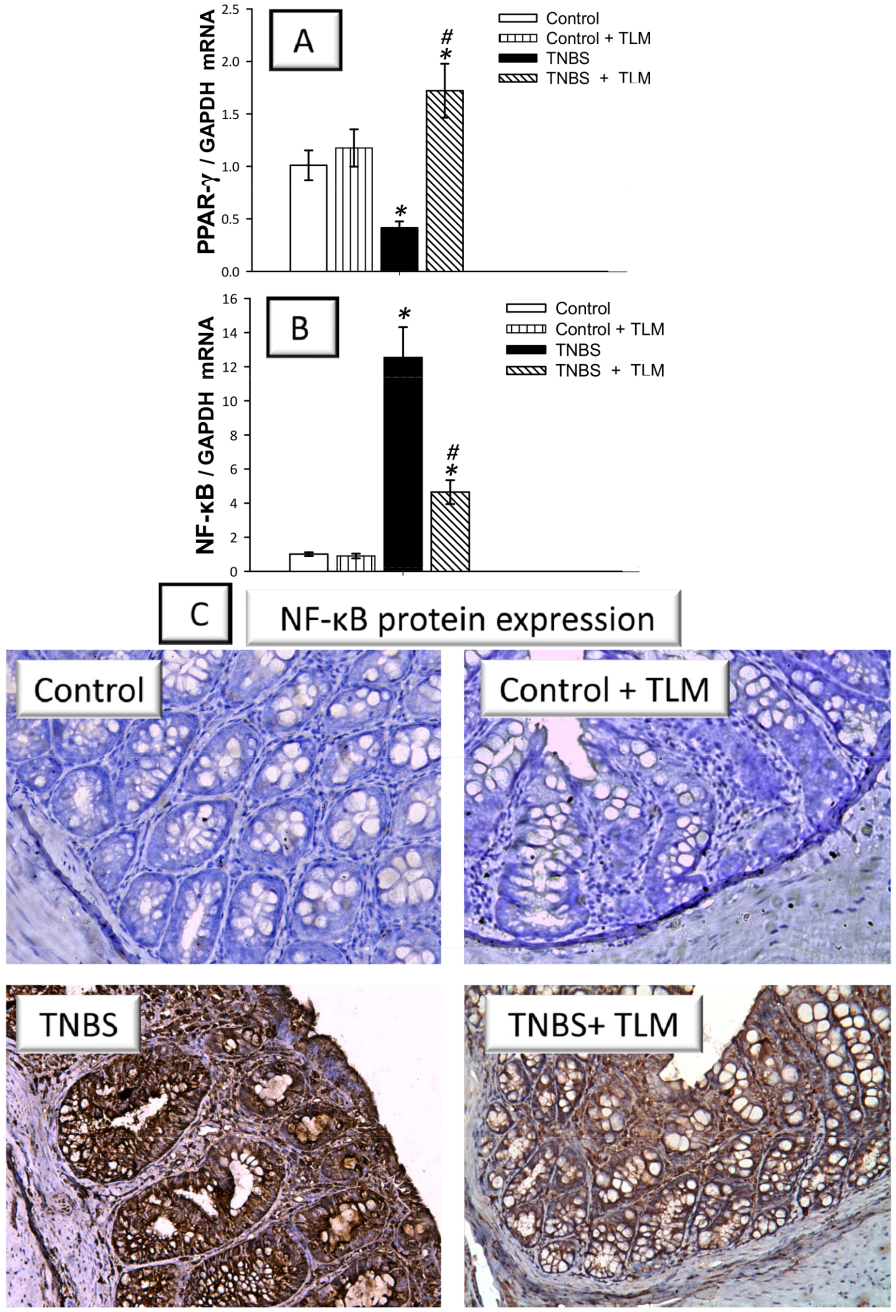
Effect of telmisartan on the mRNA expression of PPAR-γ and mRNA/ protein expression of NF-κB in the colon of rats with TNBS colitis. (A) mRNA expression of peroxisome proliferator activated receptor-gamma; PPAR-γ. (B) mRNA expression of nuclear factor kappa B; NF-κB. The mRNA expression was detected by quantitative real-time RT-PCR. Measurements were performed 5 days post TNBS instillation and TLM was administered for 12 days starting 1 week before colitis induction. Data are expressed as mean ± SD (n =  6). *Significant difference from control gp at *p*<*0.05, #* Significant difference from TNBS colitis gp at *p*<*0.05.* (C) Immunohistochemical detection of NF-κB p65 expression. Representative images for the detection of NF-κBp65 expression from colon samples harvested on the 5^th^ day post TNBS (magnification: × 200). Control and control + TLM gps: minimal expression; TNBS gp: extensive expression (brown color); TNBS+ TLM gp: attenuated expression. TLM; telmisartan, TNBS; tri-nitrobenzene sulfonic acid.

**Figure 5:**
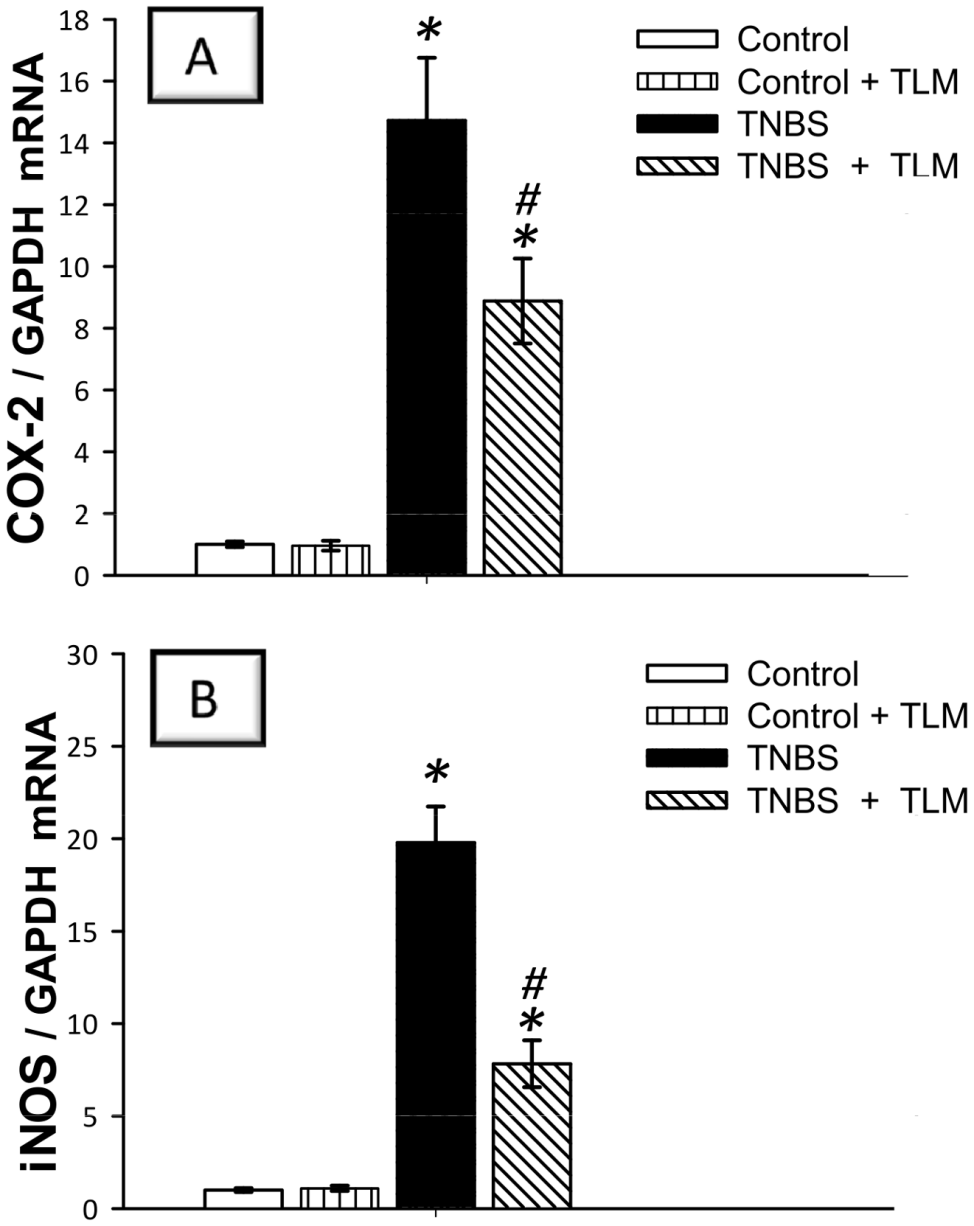
Effect of telmisartan on the mRNA expression of COX-2 and iNOS proinflammatory genes in colon of rats with TNBS colitis. (A) Cyclo-oxygenase-2; COX-2. (B) Inducible nitric oxide synthase; iNOS. The mRNA expression was detected by quantitative real-time RT-PCR. Measurements were performed 5 days post TNBS instillation and TLM was administered for 12 days starting 1 week before colitis induction. Data are expressed as mean ± SD (n = 6). *Significant difference from control gp at *p*<*0.05, #* Significant difference from TNBS colitis gp at *p*<*0.05.* TLM; telmisartan, TNBS; tri-nitrobenzene sulfonic acid.

Since the beneficial effects of TLM are partly ascribed to its PPAR-γ partial agonist properties [12], we verified its impact on the mRNA expression of PPAR-γ in colonic tissues. As depicted in Figure 4A, TNBS administration suppressed PPAR-γ (41% of the control levels) whereas TLM upregulated its levels in colonic tissues indicating a possible role of PPAR-γ in attenuation of colon inflammation. We further extended our investigation to assess the mRNA expression of NF-κB, COX-2 and iNOS which play crucial proinflammatory roles during the pathogenesis of IBD [40]. In animals with TNBS colitis, data revealed significant increase in the colonic expression of activated NF-κB p65 subunit at the mRNA level (12.5 fold) which was also confirmed by immunohistochemistry that demonstrated extensive NF-κB p65 expression (Figure 4B, C). In the same context, the mRNA expression of COX-2 and iNOS, downstream targets of NF-κB, was elevated by 14.7- and 19.8-fold increases, respectively, as compared to control rats (Figure 5). Interestingly, TLM significantly decreased the mRNA/protein expression of NF-κB p65 and the mRNA of COX-2 and iNOS, indicating that TLM downregulation of these proinflammatory genes is implicated in its beneficial protective effects against colitis.

### Telmisartan inhibits oxidative stress and enhances colon antioxidant defenses

**Figure 6:**
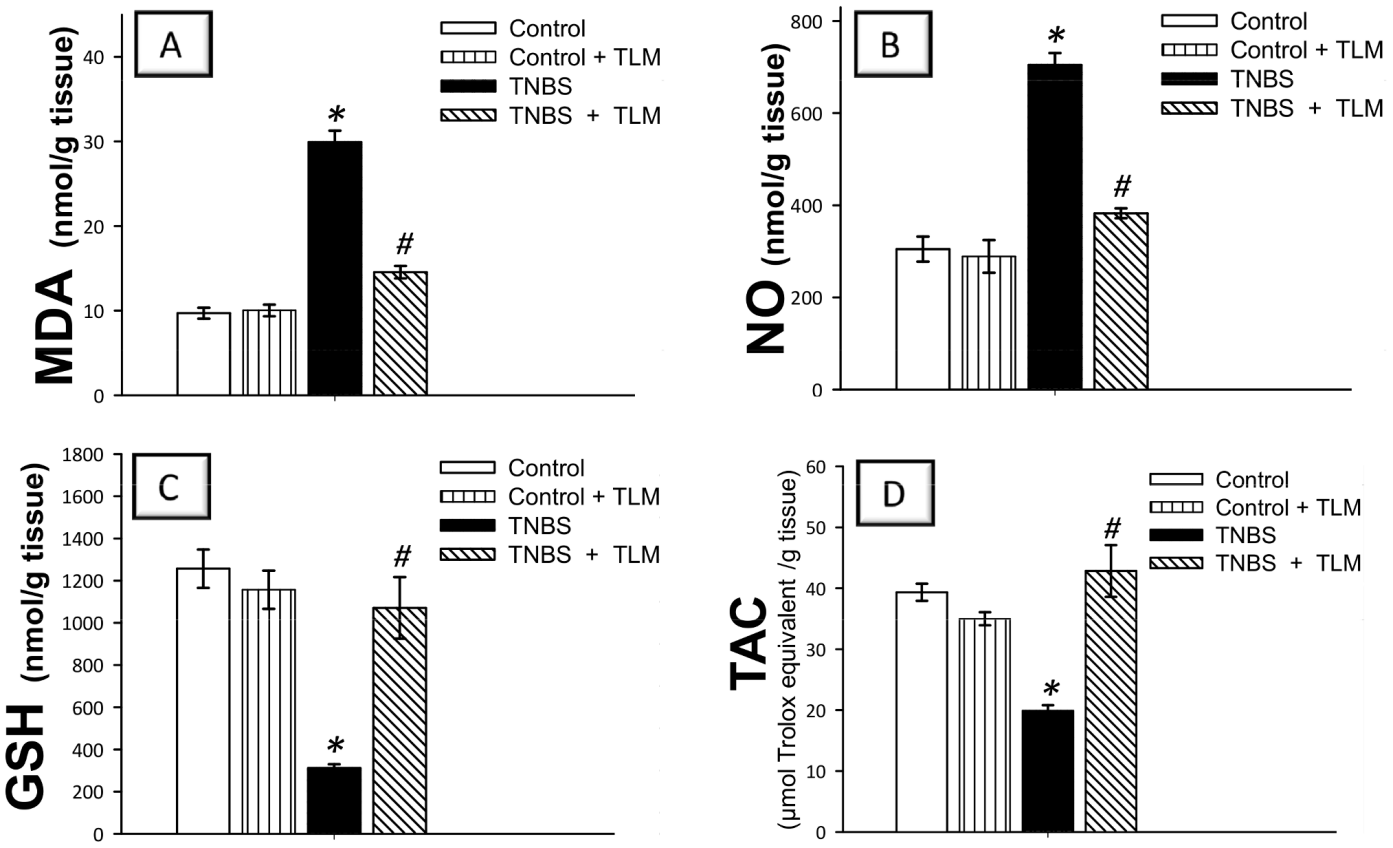
Telmisartan ameliorates oxidative stress and enhances antioxidant defenses in the colon of rats subjected to TNBS-induced colitis. (A) Lipid peroxides expressed as malondialdehyde; MDA. (B) Nitric oxide; NO. (C) Reduced glutathione; GSH. (D) Total antioxidant capacity; TAC. Measurements were performed 5 days post TNBS instillation and TLM was administered for 12 days starting 1 week before colitis induction. Data are expressed as mean ± SEM (n =  8) *Significant difference from control gp at *p*<*0.05, #* Significant difference from TNBS colitis gp at *p*<*0.05.* TLM; telmisartan, TNBS; tri-nitrobenzene sulfonic acid.

**Figure 7:**
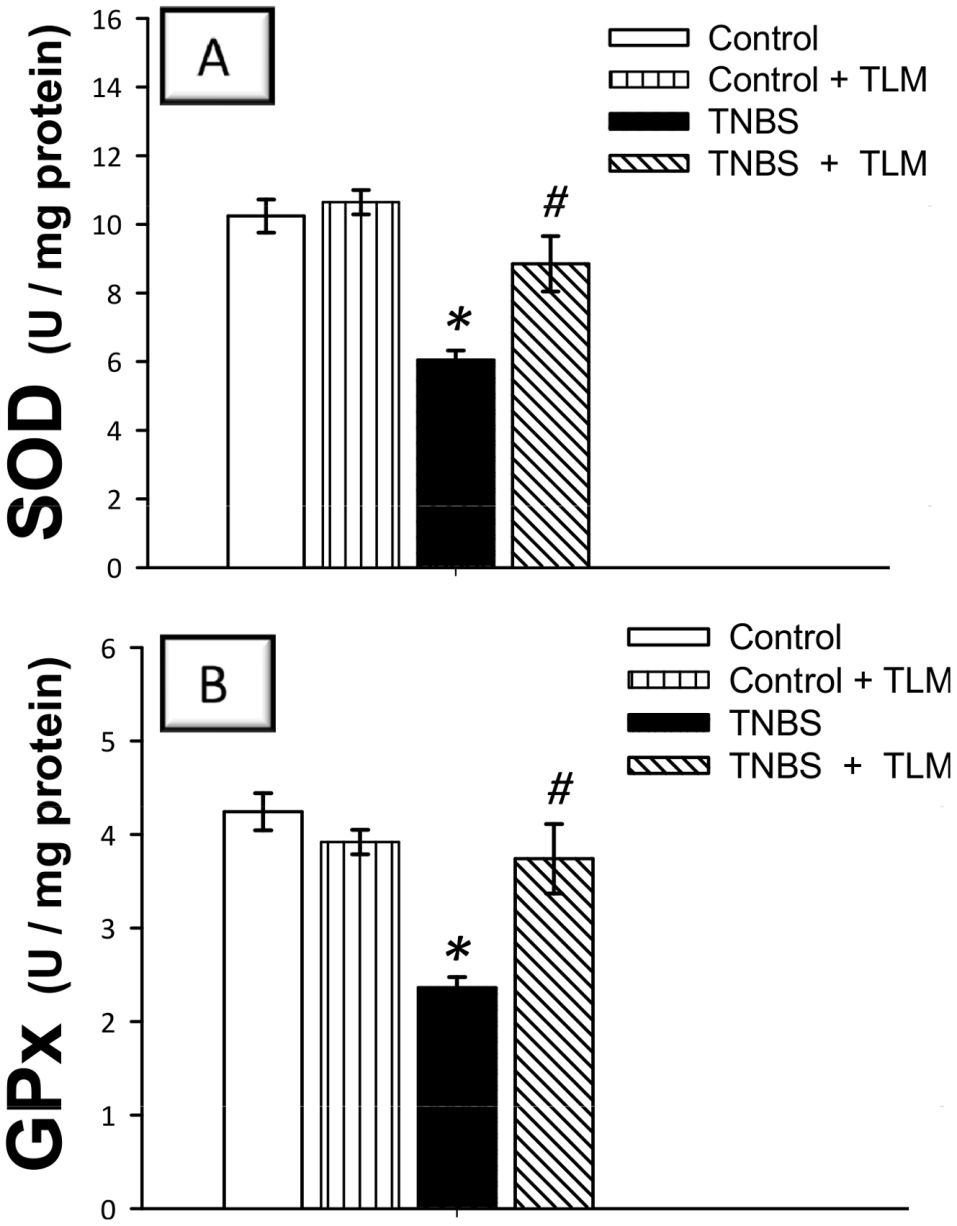
Telmisartan enhances activites of superoxide dismutase; SOD (A) and glutathione peroxidase; GPx (B) antioxidant enzymes in colon of rats with TNBS colitis. Measurements were performed 5 days post TNBS instillation and TLM was administered for 12 days starting 1 week before colitis induction. Data are expressed as mean ± SEM (n =  8) *Significant difference from control gp at *p*<*0.05, #* Significant difference from TNBS colitis gp at *p*<*0.05.* TLM; telmisartan, TNBS; tri-nitrobenzene sulfonic acid.

During the development of IBD, the inflammatory process provokes oxidative stress and diminishes cellular antioxidant capacity [3]. Instillation of TNBS resulted in a marked oxidative stress as indicated by increased levels of MDA (308%) and NO (231%) along with diminished levels of GSH (25%) and TAC (51%) and activities of SOD (59%) and GPx (56%), as compared to control group (Figures 6 and 7). Administration of TLM afforded significant protection against oxidative stress as evidenced by decrease of MDA & NO levels in addition to reinstatement of GSH & TAC levels and SOD & GPx activities, as compared to TNBS colitis group. These effects suggest that TLM attenuation of oxidative perturbations and boosting of colonic enzymatic and non-enzymatic antioxidant defenses play a role in attenuation of TNBS colitis.

### Telmisartan downregulates the mRNA expression of apoptotic genes

**Figure 8:**
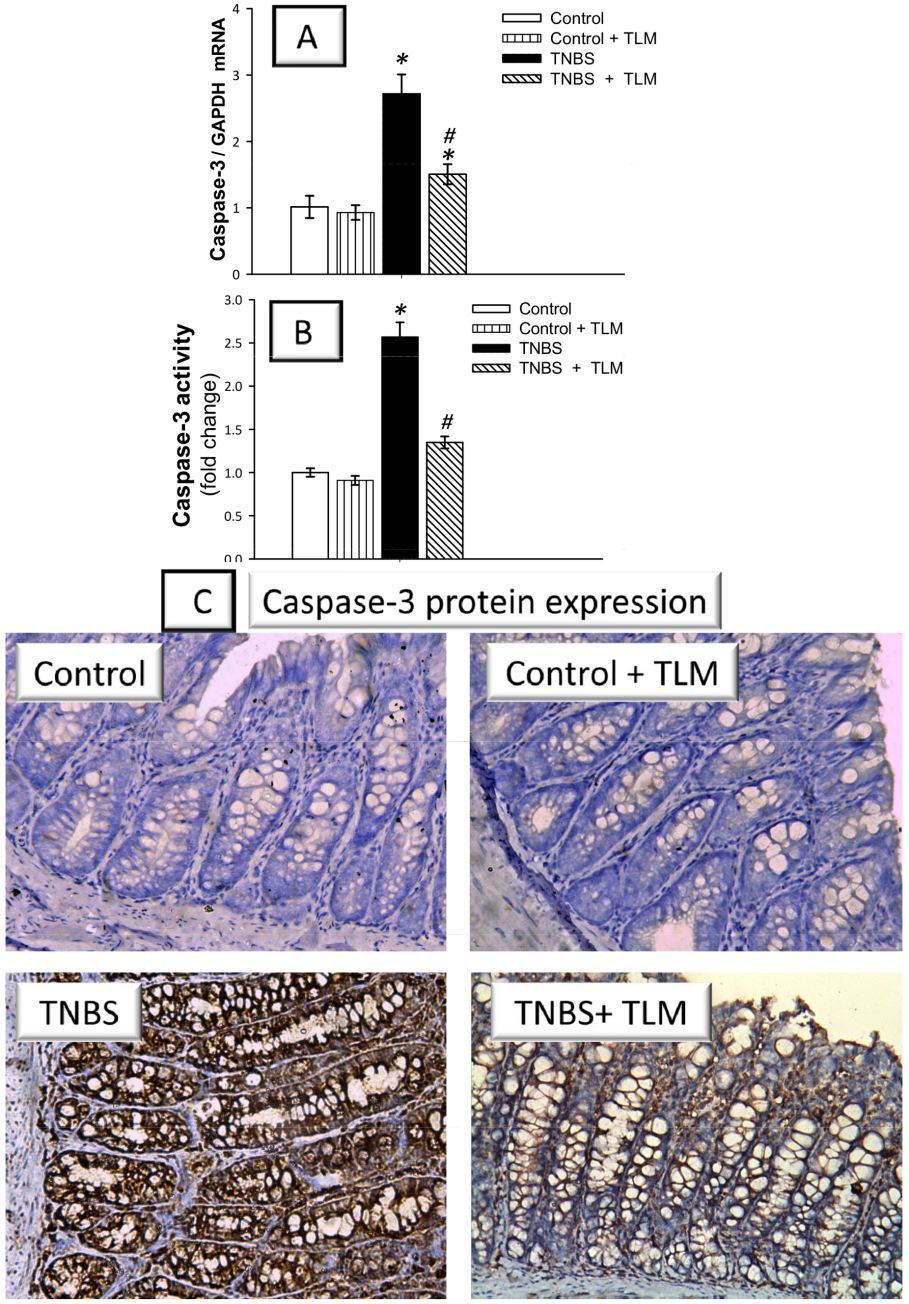
Effect of telmisartan on the mRNA expression, activity and protein expression of caspase-3 in the colon of rats with TNBS colitis. (A) Caspase-3 mRNA expression. (B) Caspase-3 activity. Measurements were performed 5 days post TNBS instillation and TLM was administered for 12 days starting 1 week before colitis induction. Caspase-3 mRNA expression was detected by quantitative real-time RT-PCR (data are expressed as mean ± SD; n =  6) and the activity was measured using ELISA (results are expressed as mean ± SEM; n = 8). *Significant difference from control gp at *p*<*0.05, #* Significant difference from TNBS colitis gp at *p*<*0.05.* (C) Immunohistochemical detection of caspase-3 protein expression. Representative images of caspase-3 expression from colon samples harvested on the 5^th^ day post TNBS (magnification: × 200). Control and control + TLM gps: minimal expression; TNBS gp: extensive expression (brown color); TNBS+ TLM gp: attenuated expression. TLM; telmisartan, TNBS; tri-nitrobenzene sulfonic acid.

**Figure 9:**
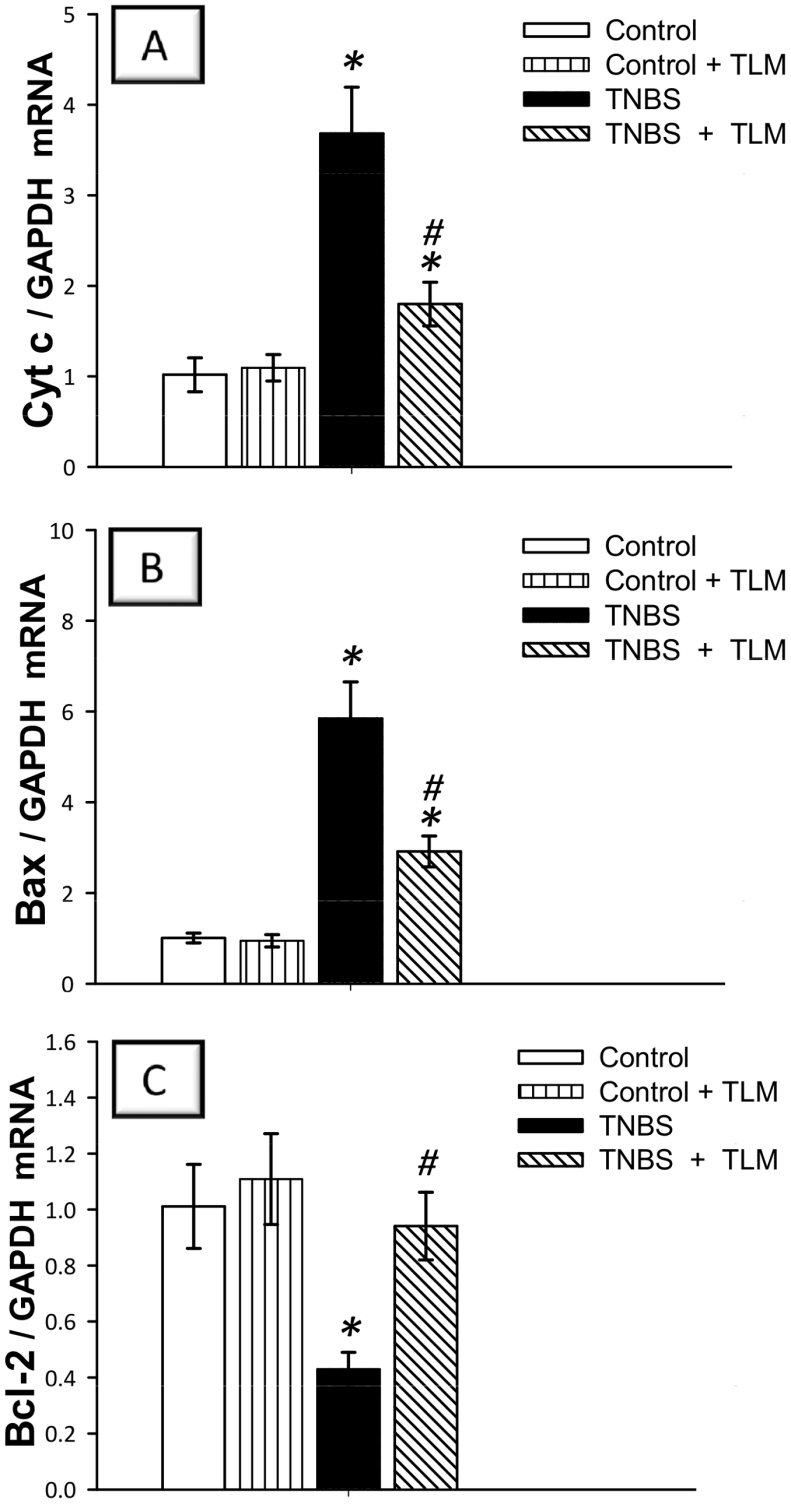
Effect of telmisartan on mRNA expression of Cyt c, Bax and Bcl-2 apoptotic genes in colon of rats with TNBS colitis. (A) Cytochrome c; Cyt c. (B) Bcl-2 associated x protein; Bax. (C) B cell lymphoma-2; Bcl-2. mRNA expression was detected by quantitative real-time RT-PCR. Measurements were performed 5 days post TNBS instillation and TLM was administered for 12 days starting 1 week before colitis induction. Data are expressed as mean ± SD (n =  6). *Significant difference from control gp at *p*<*0.05, #* Significant difference from TNBS colitis gp at *p*<*0.05.* TLM; telmisartan, TNBS; tri-nitrobenzene sulfonic acid.

During the pathogenesis of IBD, exposure of intestinal mucosa to intracellular stressors such as ROS under inflammatory stimuli triggers intestinal epithelial cell apoptosis [7], [41]. Thus, we investigated whether TLM can suppress apoptosis in colonic mucosa to protect against TNBS colitis. This was addressed via assessing the mRNA expression of cytochrome c, Bax, Bcl-2 and caspase-3. As observed in Figure 8A, instillation of TNBS triggered apoptosis of inflamed colon as indicated by a 2.7 fold increase of caspase-3 mRNA expression, a reliable indicator for apoptosis [7]. This finding was further augmented by the increased activity as well as protein expression of caspase-3 (Figure 8B, C) in TNBS colitis group. In the same context, increased mRNA expression of the pro-apoptotic cytochrome c (3.7 fold) and Bax (5.9 fold) together with downregulation of Bcl-2, an anti-apoptotic gene, were observed (Figure 9). Interestingly, administration of TLM counteracted these changes in favor of cell survival suggesting that TLM protects the colonic mucosa from apoptosis in TNBS-induced colitis.

## Discussion

**Figure 10:**
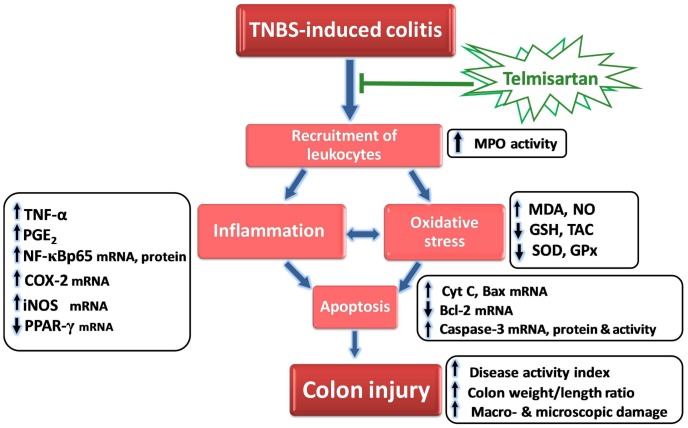
Diagram depicting the alleviating actions of telmisartan in TNBS-induced colitis.

The current study highlights the alleviating effects of TLM, an Ang II AT-1 receptor antagonist with PPAR-γ partial agonist features, in TNBS-induced colitis, an experimental model of human IBD. These beneficial effects were associated with modulation of colonic PPAR-γ, NF-κB and its downstream COX-2, iNOS and inflammatory cytokines. TLM attenuated oxidative stress and boosted the antioxidant defenses. It also downregulated colonic pro-apoptotic signals with concomitant upregulation of the anti-apoptotic Bcl-2 (Figure 10).

Besides its classical role in the regulation of blood pressure and fluid homoeostasis, novel activities of RAS have been identified including immune cell modulation with proinflammatory actions [8]. RAS has been implicated in the pathogenesis of IBD via upregulation of Ang II AT1 receptors throughout the colon [1], [8]. Ang II is involved in several key steps of the inflammatory cascade that ultimately provoke intestinal injury and ulceration including polymorphonuclear leukocyte (PMN) infiltration, probably, via upregulation of adhesion molecules [8], [42].

TNBS-induced colitis mimics human IBD with respect to several histological alterations including mucosal invasion of PMN cells as indicated by MPO which also generates hypochlorous acid and contributes to colon injury [39]. In the current study, TLM attenuated leukocyte influx to inflamed colon as revealed by histopathology and diminished MPO activity. These observations are in accord with previous studies [15], [23]. The mitigation of leukocyte influx may account for the beneficial effects of TLM against colon injury and is most likely mediated via the observed inhibition of TNF-α and oxidative stress since they trigger the expression of P-selectin, ICAM-1 and MAdCAM-1 adhesion molecules in colonic mucosa [42].

Our data also described an upregulation of the inflammatory status with increased levels of TNF-α and PGE_2_ along with NF-κB, COX-2 and iNOS in rats with TNBS colitis. These findings are consistent with previous reports [3], [10], [43], [44]. Ang II has been previously reported to increase the generation of TNF-α probably via activation of NF-κB [8]. TNF-α is a pleiotropic cytokine which has been implicated in IBD pathogenesis via activation of immune cells, generation of other proinflammatory cytokines and overexpression of angiotensinogen and Ang II [2], [8]. Furthermore, the observed increase in colonic PGE_2_ can be attributed to its enhanced synthesis via COX-2 enzyme whose expression is upregulated by Ang II [8]. Our data also revealed increased colonic levels of the anti-inflammatory IL-10. Previously, upregulation of IL-10 has been reported in plasma of patients with IBD [4] and colons of rats with TNBS- and dextran sulfate-induced colitis [5], [6]. Increased circulating levels of IL-10 can be envisioned as a compensatory mechanism against colonic injury and is thought to play a role in limiting mucosal inflammation since IL-10 downregulates MHC class II antigen presentation and subsequent release of pro-inflammatory cytokines [45]. However, the increased IL-10 levels might not be adequate to fully control colon inflammation due to low IL-10 bioavailability [46].

Ang II has been reported to activate several nuclear transcription factors including NF-κB which is also driven by ROS and inflammatory cytokines [3], [8]. The NF-κB regulates the expression of several proinflammatory genes including TNF-α, COX-2 and iNOS that play key roles in IBD and TNBS colitis [8]. NF-κB, a heterodimer of p65 and p50 subunits of Rel protein family, is retained in inactive state via association with the inhibitory protein IκBα in the cytosol. Upon exposure of cells to stress conditions, activation of NF-κB is triggered via phosphorylation and proteasomal degradation of IκBα which liberates NF-κB that translocates to the nucleus to control the expression of target genes [40]. In the current study, the mRNA and the protein expression of activated NF-κB p65 subunit were elevated in rats with TNBS colitis. This finding is in agreement with previous studies [40], [47]. Our data also revealed enhanced levels of COX-2 and iNOS which are downstream targets of NF-κB. COX-2 generates an arsenal of PGE_2_ and TXB_2_ which provokes intestinal hyperemia and edema whereas iNOS activation releases a surplus of NO which undermines colon integrity via synthesis of peroxynitrite, a potent oxidizing agent which is formed via reaction of NO with superoxide anion [48].

Interestingly, TLM increased the levels of PPAR-γ with concomitant suppression of colon NF-κBp65, COX-2, and iNOS along with TNF-α, PGE_2_ and NO. Similar findings have been reported for TLM in autoimmune myocarditis [49], stroke [20] and renal oxidative damage [50]. Upregulation of TLM to PPAR-γ has been reported to suppress the production of inflammatory mediators, at least partly, via inhibition of NF-κB [21]. The observed inhibition of NF-κB together with its downstream effectors as COX-2, iNOS and TNF-α is regarded as an advantage in the management of IBD [47]. Since the promoter regions of COX-2, iNOS and TNF-α contain consensus binding motifs for NF-κB, it would be conceivable to understand that downregulation of these targets is secondary to NF-κB inhibition by TLM [49]. TLM also attenuated PGE_2_ and NO levels, an effect probably linked to inhibition of COX-2 and iNOS enzymes, respectively [3]. The observed restoration of IL-10 by TLM probably reflects improvement of the inflammatory status which was associated with suppression of proinflammatory signals. In this context, the observed TLM lowering of TNF-α may be implicated in the mitigation of colonic IL-10 levels, since the release of IL-10 is driven by elevated levels of proinflammatory cytokines [45]. Thus, the current data reinforce the alleviating actions of TLM in TNBS colitis owing to its pleiotropic anti-inflammatory actions.

The implication of oxidative stress in the pathogenesis of IBD has been highlighted by several clinical [51] and experimental studies [1] where the surge of ROS and NO generated by activated neutrophils and macrophages inflicts intestinal injury. Ang II has been reported to trigger oxidative stress with generation of superoxide anions via NADH/NADPH oxidase in addition to hydrogen peroxide and hydroxyl radicals [8], [52]. In the current study, enhanced oxidative stress was verified by increase in lipid peroxides & NO with concomitant decrease of GSH & TAC levels and SOD & GPx activities in TNBS-induced colitis. These observations are in line with previous studies [3], [10], [43], [44].

In the current study, TLM combated oxidative stress and boosted the antioxidant status in animals with TNBS colitis as evidenced by reduction of MDA and NO levels in addition to reinstatement of GSH &TAC levels and SOD & GPx activities. These findings are in agreement with previous studies and they reinforce the premise that the antioxidant properties of TLM are implicated in alleviation of TNBS colitis [15], [23], [43], [49]. The antioxidant features of TLM have been ascribed to scavenging hydroxyl radicals via its benzimidazolic and benzoic moieties [53] in addition to downregulation of NADPH oxidase subunits [14], [23]. Interestingly, Fujita et al. [50] demonstrated that TLM inhibited renal oxidative stress in diabetic mice via upregulation of Nrf2 and SOD. The observed preservation of GSH, TAC and SOD & GPx antioxidant enzymes signifies the role of TLM in boosting colonic antioxidant defenses and correlates well with the reported preservation of endogenous antioxidants in experimental myocardial infarction [15].

Our results also described an *in vivo* activation of apoptosis in colonic tissues as indicated by upregulation of cytochrome c, Bax and caspase-3 pro-apoptotic genes along with downregulation of the anti-apoptotic Bcl-2. These data are in concert with previous literature [47], [54]. Evidence has highlighted the pro-apoptotic effects of Ang II which were totally abolished by Ang II neutralizing antibodies and ARBs [55]. The increased apoptosis of epithelial cells likely results in alteration of the epithelial barrier, thereby contributing to intestinal injury [7]. Elevated rate of colonic apoptosis has been reported in patients with UC and TNBS colitis [54], [56]. It has been reported that the oxidative stress triggers the expression of several genes responsible for cellular death by apoptosis [54]. Apoptosis is regulated, in part, by the Bcl-2 family including Bcl-2 and Bax. Bcl-2 is regarded as a prosurvival signal whereas Bax is a pro-apoptotic member since it binds and antagonizes the effects of Bcl-2 [7]. Increased Bax/Bcl-2 ratio enhances the release of cytochrome c from mitochondria to cytosol, which activates caspase-9 and ultimately caspase-3, the major executioner caspase [7], [54].

Our data revealed that TLM upregulated Bcl-2 with downregulation of the pro-apoptotic cytochrome c, Bax and caspase-3, indicating attenuation of colonic apoptosis. These findings are consistent with previous reports that described the inhibition of TLM to apoptosis in autoimmune myocarditis [49] and testis of diabetic rats [21]. The attenuation of colonic apoptosis can be ascribed to the observed inhibition of oxidative stress since excessive exposure of intestinal mucosa to ROS under inflammatory stimuli enhances epithelial apoptosis [41]. Besides, the observed elevation of PPAR-γ is likely engaged in apoptosis suppression as suggested by previous studies for TLM [15], [23] and for PPAR-γ agonists such as rosiglitazone [57].

During colonic inflammation, several proinflammatory cytokines such as INF-γ, IL-1β and IL-8 besides anti-inflammatory cytokines as IL-4 play a major role in the pathogenesis of IBD and, thus, their investigation can delineate molecular aspects of TLM protective actions [58]. Our future studies will focus on investigating these targets in order to precisely elucidate the underlying molecular mechanisms for TLM in IBD. In our experiments, while several molecular aspects of inflammation and apoptosis were examined at the level of mRNA, some of these parameters were confirmed by the protein expression as in case of NF-κB p65 and caspase-3 that also revealed good correlation with the mRNA data. Additionally, at both mRNA and protein expression levels, elevation of COX-2 and iNOS along with suppression of PPAR-γ have been previously reported in experimental IBD models [59], [60]. In these studies, the effective treatment mitigated the expression of these genes at both mRNA and protein levels [59], [60]. Previously, decrease in the mRNA levels of proinflammatory genes have been regarded as an early sign of suppressed inflammatory signaling [60]. Yet, the mRNA and protein expression are differently expressed, probably due to post-translational modification of mRNA [61]. Thus, upcoming investigation of the molecular events at both mRNA and protein levels will precisely delineate the underlying mechanisms for TLM actions in experimental IBD.

## Conclusions

In conclusion, the current study highlights evidences for the promising protective effects of TLM in TNBS-induced colitis, an experimental model of IBD. These favorable actions were linked with modulation of PPAR-γ, NF-κB and its downstream COX-2, iNOS and inflammatory cytokines. TLM mitigated oxidative perturbations and boosted enzymatic/non-enzymatic antioxidant defenses. Besides, it downregulated colonic pro-apoptotic genes with concomitant upregulation of Bcl-2. Among available ARBs, TLM displays the strongest binding to AT1 receptors, the longest half-life and high lipophilicity besides its partial PPAR-γ agonist properties [12]. Together, the current study suggests the beneficial effects of TLM in experimental IBD. Further studies are warranted to investigate the potential therapeutic efficacy of TLM in the management of IBD following the manifestation of symptoms. Besides, the exact molecular mechanisms and signaling networks implicated in TLM actions need to be identified.
